# Artificial Urinary Sphincter Is Better Than Slings for Moderate Male Stress Urinary Incontinence With Acceptable Complication Rate: A Systematic Review and Meta-Analysis

**DOI:** 10.3389/fsurg.2022.841555

**Published:** 2022-02-09

**Authors:** Lede Lin, Wenjin Sun, Xiaotong Guo, Liang Zhou

**Affiliations:** ^1^Department of Urology, Institute of Urology (Laboratory of Reconstructive Urology), West China Hospital, Sichuan University, Chengdu, China; ^2^Department of General Practice, West China Hospital, Sichuan University, Chengdu, China; ^3^Department of Thoracic Oncology, West China Hospital, Sichuan University, Chengdu, China

**Keywords:** artificial urinary sphincter, slings, male stress urinary incontinence, systematic review, meta-analysis

## Abstract

**Background:**

This meta-analysis aimed to compare the efficacy of artificial urinary sphincter (AUS) and slings for the treatment of moderate male stress urinary incontinence (SUI) based on existing data.

**Methods:**

The study was in accordance with the Preferred Reporting Items for Systematic Reviews and Meta-Analysis. We searched the widely acknowledged database including PubMed, Embase (Ovid version), Medline (Ovid version), and Cochrane Central Register of Controlled Trials (till February 2021). Male patients with moderate SUI who underwent AUS or slings procedure over 18 years old and had been monitored with a mean follow-up time of at least 12 months were included. The primary outcome was success rate defined as daily pad use with 0–1 pad/d postoperatively. Articles with congruent outcomes were suitable for inclusion. The secondary outcome included complication rate of infection, erosion, acute urinary retention, and surgical revision.

**Results:**

Five studies with a total of 509 patients (295 for slings and 214 for AUS) were recruited. The success rate was higher in AUS with an odds ratio (OR) = 0.57 (95% CI: 0.36–0.90). As for the overall complication rate, no significant difference was discovered between slings and AUS groups (OR = 1.06, 95% CI: 0.58–1.92, *P* = 0.86).

**Conclusion:**

To conclude, AUS was better than slings for moderate male SUI treatment according to daily pad use with an acceptable complication rate. The slings also had clinical value and were options when aging male patients were AUS naive and refused inserted mechanical devices. High-quality pieces of evidence are needed to confirm the efficacy of AUS and slings in moderate male SUI.

**Systematic Review Registration:** https://www.crd.york.ac.uk/prospero/display_record.php?RecordID=271203, identifier: CRD42021271203.

## Introduction

Male stress urinary incontinence (SUI) is not a rare disease and is recognized to have a negative impact on the patients' quality of life. It has been reported that the prevalence of SUI in male patients increased with age, with a proportion of around 2 and 4% for 48–64 years old and over 65, respectively (1). As for the etiology of SUI, postprostatectomy SUI has caused significant attention, which is the second most common complication after radical prostatectomy, with moderate-to-severe SUI accounting for an estimated proportion of 10–20% male patients (2–4).

Two main solutions are recommended for the treatment of male SUI after the failure of conservative therapy: artificial urinary sphincter (AUS) and slings. Up till now, AUS is still recognized as the gold standard for the treatment of moderate-to-severe postprostatectomy SUI, (5) for its satisfactory cure rate is usually more than 80% utilizing the strict definition of cure as 0–1 pad per day (6–10). However, more and more urologists put their focus on slings for the treatment of mild-to-moderate SUI, as a result of the easier procedure and avoiding an inserted prosthesis (11). On the other hand, the patients also put a priority to slings over AUS based on their preference (12).

Some studies have explored the clinical outcome of postprostatectomy SUI between AUS and slings, but the results are confusing (7–10, 13–15). Hence, researches on meta-analysis is urgently needed to integrate the existing evidence to draw a conclusion about the preference of two SUI treatments mentioned above. Nevertheless, it is regrettable that recent systematic reviews merely include network meta-analysis that indirectly compares AUS intervention or adjustable slings procedure with non-intervention group (16), or includes meta-analysis that compares the pad use per day before and after SUI surgery (17). Moreover, they did not pay attention to moderate male SUI, whose treatment seems to be a choice between AUS and slings. As a consequence, we aim to search the literature, conduct a meta-analysis, and compare the efficacy of AUS and slings for the treatment of moderate male SUI based on existing data.

## Materials and Methods

The study was in accordance with the Preferred Reporting Items for Systematic Reviews and Meta-Analysis (PRISMA). The work was registered in PROSPERO with registration number CRD42021271203.

### Search Strategy

We searched the widely acknowledged database including PubMed, Embase (Ovid version), Medline (Ovid version), and Cochrane Central Register of Controlled Trials (till February 2021). The keywords were described as artificial urinary sphincter and sling and urinary incontinence.

### Inclusion and Exclusion Criteria

The articles were eligible if they contained the comparison of AUS and slings for the treatment of moderate male SUI in patients above 18 years old. As not all studies reported 24 h pad test results, we discussed and decided that the degree of moderate male SUI was defined as overall pad use ≤ 5 pad/d. The mean overall follow-up for both AUS and slings groups was required to be at least 12 months. Cohort study, case-controlled study, and randomized controlled study were all included. Reviews, guidelines, systematic reviews, and meta-analyses were excluded. Conference articles, editorial comments, protocols, and cases involved with pediatric patients were also excluded. The article language was restricted to English, and the articles with inadequate follow-up of <12 months in either the AUS group or slings group were excluded.

### Study Outcomes

The primary outcome was surgical success defined as daily pad use of 0–1 pad/d postoperatively. The articles with congruent outcomes reporting were suitable for inclusion, for example, some articles described success as ≤ 1 pad/d postoperatively for patients requiring ≥2 pad/d preoperatively, and 0 pad/d for those requiring 1 pad/d preoperatively (14). The secondary outcome included complication rate of infection, erosion, and acute urinary retention. For the articles missing critical data or with the subjective outcome from patients' perspectives, we decided not to include them in the final analysis.

### Data Extraction

We screened the title and abstract to identify eligible references, and then assessed the full text to determine the ultimate selection for qualitative and quantitative analysis. A discrepancy was carefully discussed and resolved when met.

Based on the literature available online, our team determined to collect data as follows: first author, years of publication, country, study design, patients' selection, outcome definition, number of cases for each group, mean age for each group, mean preoperative and postoperative pad used daily for each group, mean follow-up time for each group, success rate for each group, and complication rate for each group. For the articles reporting median data, we tried to convert them to mean data as possible as we could to retain the accuracy of raw data, in accordance with feasible methods (18, 19).

### Risk of Bias for Articles in the Meta-Analysis

We assessed the risk of bias for articles eligible in the final meta-analysis, which was modified from the Newcastle-Ottawa Quality Assessment Scale (NOS), including representativeness of the cohort, ascertainment of intervention, documentation that outcome of interest was not present at the start of the study, comparability of cohorts on the basis of the design or analysis, assessment of outcomes, follow-up long enough for outcomes to occur, and adequacy of follow-up of cohorts. After reviewing the full-text carefully, low risk of bias, high risk of bias, and unclear risk of bias were applied to each eligible article according to stated information. All the procedure was completed on RevMan 5.3 (Cochrane, London, UK).

### Data Analysis

The meta-analysis was operated on RevMan 5.3 (Cochrane, London, UK), with an odds ratio (OR) using Mantel-Haenszel statistical method for dichotomous data. The pooled results were reported as OR with 95% CI. The heterogeneity test was completed by Chi^2^ and *I*^2^-tests. Chi^2^ test with *P* > 0.10 and *I*^2^ <50% were thought to have acceptable heterogeneity. If so, it was reliable to utilize a fixed-effect model for meta-analysis and there was no need to exert a subgroup or sensitivity analysis. Otherwise, we would use a random effect model. As for the overall effect, the *Z*-test was used to certify the statistical significance, which was defined as *P* < 0.05. The results were presented as a forest plot.

### Publication Bias

The publication bias was evaluated through a funnel plot using RevMan 5.3 (Cochrane, London, UK). Ideal results were presented as symmetrical spots distribution along the central axis.

## Results

### Articles Selection

Initially, 909 articles were included after we searched the designated databases. Considering the inclusion and exclusion criteria for the study, 520 articles were screened and 303 full-texts were assessed carefully for eligibility. Finally, 5 eligible articles were selected in meta-analysis (7–9, 14, 15). The flow diagram of articles selection was described in Supplementary Figure 1.

### Characteristics of Included Studies

Five studies with a total of 509 patients (295 for slings and 214 for AUS) were recruited. Two were in Korea, 1 in the USA, and the other countries were Canada and Italy. The included patients' criteria varied slightly among studies. Four presented with moderate SUI and the other one included postprostatectomy incontinence with a mean pad use of 4.8 per day. Surgical success definition and other detailed information were illustrated in Table 1. We found that generally, all studies defined surgical success as daily pad use with 0–1 pad/d. One study included male patients according to the Male Stress Incontinence Grading System (MSIGS) with moderate scores. There were various types of slings in studies, including AdVance, AdVanceXP, Augus, TiLOOP, and so on.

**Table 1:** Characteristics of included studies.

**Author**	**Period**	**Nation**	**Inclusion criteria**	**Outcome definition**	**Cases for slings**	**Cases for AUS**	**Mean age for slings**	**Mean age for AUS**	**Baseline comparability**
Hoy	August 2004–March 2013	Canada	Mild to moderate PPI (≤ 5 pad/d)	Continence: ≤ 1 pad/d post-operatively for patients requiring ≥2 pad/d pre-operatively, and 0 pad/d for those requiring 1 pad/d pre-operatively	76	48	66.2	68.1	Low risk
Lim	January 2009–June 2013	Korea	Moderate PPI (2–4 pad/d)	Success: 0–1 pad/d	20	13	70.9	73.5	Low risk
Kim	November 2001–December 2016	Korea	Received AUS or adjustable male slings because of PPI	Success: 0–1 pad/d	50	53	70.8	69.1	High risk^#^
Khouri	2008–2019	USA	Men presenting with moderate SUI (MSIGS scores of 2–3)	Failure: >1 pad/d or the need for subsequent incontinence procedure	114	65	66.5	70.8	Low risk
Sacco	July 2011–December 2017	Italy	Moderate (3–5 pad/d) stress-prevalent PPI	Cure: 0–1 pads/d	35	35	69.64^*^	70.64^*^	Low risk

*AUS, artificial urinary sphincter; PPI, postprostatectomy incontinence; SUI, stress urinary incontinence; MSIGS, the Male Stress Incontinence Grading System*.

* *Articles reported raw data in median type and we converted them to mean data as possible as we could to retain the accuracy of raw data*.

# *Daily pad use was significantly higher in the AUS group than that in the slings group*.

### Risk of Bias for Included Articles

The risk of bias explanation was detailed in Supplementary Figures 2, 3. Only one article had a high risk of baseline comparison between the groups while other items were all with low risks. The article with one high-risk item was due to the overall mean pad use of 4.8 pad/d, which was nearly 5 pads per day.

### Primary Outcome

**Figure 1:**
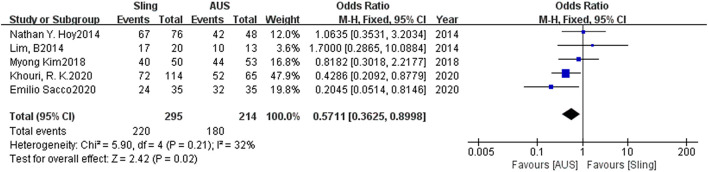
Forest plot of comparison of success rate for slings vs. AUS. AUS, artificial urinary sphincter.

The success rate yielded a statistically significant outcome in a fixed-model analysis, with pooled OR of 0.57 (95% CI: 0.36–0.90). Heterogeneity was permissible and *P*-value for the overall effect was far lower than 0.05, indicating that the model was authenticated. As for the heterogeneity *I*^2^ =32%, we considered a fixed model was appropriate and there was no need to conduct subgroup analysis or sensitivity analysis. The Forest plot was shown in Figure 1.

### Secondary Outcome

**Figure 2:**
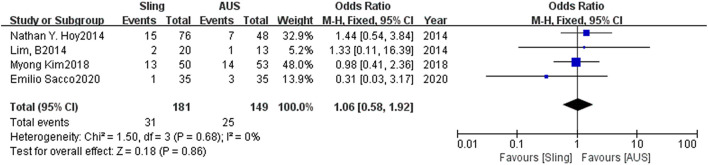
Forest plot of comparison of complication rate for slings vs. AUS. AUS, artificial urinary sphincter.

Among the 5 included articles, one had not made clear what kind of complication happened in both the slings and AUS groups. As we only included the above-mentioned complication of infection, erosion, and acute urinary retention, finally 4 articles were included in further analysis, which was depicted in Figure 2. Totally, infection happened in 8 and 10 patients for slings and AUS groups, respectively, with erosion in 5 and 11, and acute urinary retention in 18 and 4 for slings and AUS groups, respectively. Figure 2 showed no significant difference was discovered between slings and AUS groups (OR = 1.06, 95% CI: 0.58–1.92, *P* = 0.86) with acceptable heterogeneity. The subgroup analysis for specific complication types were detailed in Supplementary Figures 4–6. The OR for infection, erosion, and acute urinary retention were 0.67 (95% CI: 0.27–1.66), 0.45 (95% CI: 0.16–1.26), and 2.46 (95% CI: 0.31–19.45), respectively.

### Publication Bias

The publication bias was shown in the funnel plot (Supplementary Figure 7). Roughly, the spots were distributed along the central axis.

## Discussion

In this study, we discovered that the success rate of AUS was significantly higher than that of slings, while the overall complication rate was comparable between the two groups. Considering the gold standard of AUS, we repeatedly certified its role in the treatment of moderate male SUI with appreciable success rate and acceptable complication.

Artificial urinary sphincter, after over 30 years of introduction, has shown pronounced results based on the long period of experience and a great deal of evidence (5). However, the gold standard position of AUS has been challenged because of its surgical revision rate, at around 23% (6). Linder BJ reported that the AUS revision rate was 26, 43, and 59% in 5, 10, and 15 years, respectively (20). Recently, great interest has increased in male slings due to its simple surgery procedure, low rate of adverse events, and absence of inserted operated device (11). Nevertheless, there has not been so long since its emergence, we were only able to evaluate the short-to-medium outcome of male slings. Hence, a comparison between AUS and male slings is needed.

Several articles have also explored the effect of slings and AUS on the treatment of male SUI. Chen YC evaluated the efficacy of male slings and AUS for postprostatectomy SUI, focusing on daily pad use, cure rate, and so on (17). It is frustrating that the study only compared the efficacy of both interventions before and after surgery and found that the two procedures contributed to decreased daily pad use and quality of life improvement. Guachetá Bomba PL determined the effectiveness of adjustable slings vs. AUS in patients with severe postprostatectomy SUI, despite the fact that he could only conduct a network meta-analysis to compare the two interventions indirectly (16). He concluded that both were able to reduce incontinence and improve life quality; however, the difference of effectiveness for adjustable slings vs. AUS was not significant. It was recommended by the European Association of Urology (EAU) guideline that AUS should be used for severe postprostatectomy incontinence (21) because severe male SUI did respond negatively to slings procedure (22–24). As a result, more clinicians preferred slings when it came to male patients with mild-to-moderate SUI (25). However, such patients were in gray zones where evidence was not sufficient to make a priority conclusion between AUS and slings. Thus, we conducted this study to fill in the blank of priority in the efficacy of slings and AUS in moderate male SUI based on daily pad use and complication rate.

Recently, an article came out and published its outcomes in a randomized control design (MASTER) (26). It enrolled male patients with bothersome SUI at least 12 months after prostate surgery. The inclusion and exclusion criteria generally met moderate male SUI. However, the authors utilized self-reported outcomes regarding the success rate of slings or AUS surgery, which was relatively a subjective outcome and not consistent with other included studies. Thus, we finally decided not to add the study to the analysis.

To our acknowledgment, this is the first meta-analysis to directly compare the efficacy of slings vs. AUS based on daily pad use and complication including infection, erosion, acute urinary retention, and surgical revision. We drew a conclusion that, after integrating all available evidence, AUS turned out to show a considerable success rate without increasing complication rate significantly. The funnel plot did not show obvious publication bias, although the number of eligible studies was small. Still, from the available pieces of evidence, we believed the results illustrated a trend that AUS had a priority over slings in the clinical practice of treating moderate male SUI.

Among the 5 included studies, we discovered that one article had the risk of baseline comparability, which was because of mean overall daily pad use close to 5 pads/d, which is a generally accepted boundary between moderate and severe SUI. We considered a mean follow-up time of 12 months would be appropriate to observe the mid-term outcomes of AUS and slings procedures. In addition, as there were varieties of sling types, we described the specific name of sling types, including two articles utilizing adjustable slings. We did not think readjustment was a complication for adjustable slings because it was easy to handle and patients usually had good compliance. Hence, we only included complications mentioned above, which were common for both AUS and slings.

On the other hand, some patients feared implanted mechanical devices and tried to avoid AUS operation. Taking the situation into account, we might recommend sling as an option. Alwaal reported slings-related complication in 30 days was lower than that of AUS (2.8 vs. 5.1%, *P* = 0.046) (27). Angelish Kumar surveyed the preference of male slings and AUS in patients with postprostatectomy SUI and found that when both procedures were feasible, 92% of patients would like to choose slings rather than AUS (12). The slings would also be suitable for aging male patients who had moderate SUI and were not able to suffer from AUS. This left more options after surgical failure, like sling explantation due to complications, and allowed for longer sufficient treatment. Although in our study the overall complication rate was not significantly different, we excluded subjective complications, such as perineal pain, which varied greatly among patients according to their susceptibility and lacked objective indicators. Moreover, the complication rate of infection and erosion tended to favor the slings group. From this point of view, the slings also had clinical value when patients refused or were unable to undergo an AUS intervention.

It is of great regret that our meta-analysis only included cohort studies and lacked evidence of randomized controlled studies. We tried to assess all the eligible studies with the tool of NOS, which was standardized and widely used for non-randomized controlled studies. The assessment outcomes showed that most articles were well-designed and had little risk of bias. The included patients in each study differed slightly, some based on moderate daily pad use (9, 14, 15), while some based on moderate scale score (8). Outcome variabilities measured in studies were also found, most defining surgical success as 0–1 pad/d postoperatively. All the variabilities in inclusion criteria and outcome definition were acceptable and might have little influence on our analysis because we minimized the differences among all the included articles. We do suggest further studies with high-level evidence to compare the efficacy of slings and AUS in moderate male SUI, based on specified inclusion criteria and consistent outcome definition.

To conclude, AUS was better than slings in moderate male SUI with an acceptable complication rate in our study. The slings also had clinical value and were options when aging male patients were AUS naive and refused inserted mechanical devices. More evidence with higher quality is needed to confirm the efficacy of AUS and slings in moderate male SUI.

## Data Availability Statement

The original contributions presented in the study are included in the article/Supplementary Material, further inquiries can be directed to the corresponding author/s.

## Author Contributions

LL, WS, and XG: conception and design of the study and acquisition of data. LL: data analysis and/or interpretation. LL and LZ: drafting of the manuscript and/or critical revision. LZ: approval of a final version of manuscript. All authors contributed to the article and approved the submitted version.

## Funding

The work was supported by the 1.3.5 project for disciplines of excellence, West China Hospital, Sichuan University (ZY2016104).

## Conflict of Interest

The authors declare that the research was conducted in the absence of any commercial or financial relationships that could be construed as a potential conflict of interest.

## Publisher's Note

All claims expressed in this article are solely those of the authors and do not necessarily represent those of their affiliated organizations, or those of the publisher, the editors and the reviewers. Any product that may be evaluated in this article, or claim that may be made by its manufacturer, is not guaranteed or endorsed by the publisher.

## Supplementary Material

Supplementary Figure 1 Flow diagram of articles selection.

Supplementary Figure 2 Risk of bias graph for included studies.

Supplementary Figure 3 Risk of bias summary for included studies.

Supplementary Figure 4 Forest plot of comparison of infection rate for slings vs. Aus. AUS, artificial urinary sphincter.

Supplementary Figure 5 Forest plot of comparison of erosion rate for slings vs. AUS. AUS, artificial urinary sphincter.

Supplementary Figure 6 Forest plot of comparison of acute urinary retention rate for slings vs. AUS. AUS, artificial urinary sphincter.

Supplementary Figure 7 Funnel plot of comparison of success rate for slings vs. AUS.

## Abbreviations

AUS: Artificial urinary sphincter
SUI: Stress urinary incontinence
OR: Odds ratio
CI: Confidence interval
PRISMA: The Preferred Reporting Items for Systematic Reviews and Meta-Analysis
NOS: Newcastle-Ottawa Quality Assessment Scale
MSIGS: The Male Stress Incontinence Grading System
EAU: European Association of Urology.
