# Supplementary figures and images for: Artificial Urinary Sphincter Is Better Than Slings for Moderate Male Stress Urinary Incontinence With Acceptable Complication Rate: A Systematic Review and Meta-Analysis
Source: Front Surg. 2022 Feb 9;9:841555. doi: 10.3389/fsurg.2022.841555 (PMC8863861; doi:10.3389/fsurg.2022.841555)

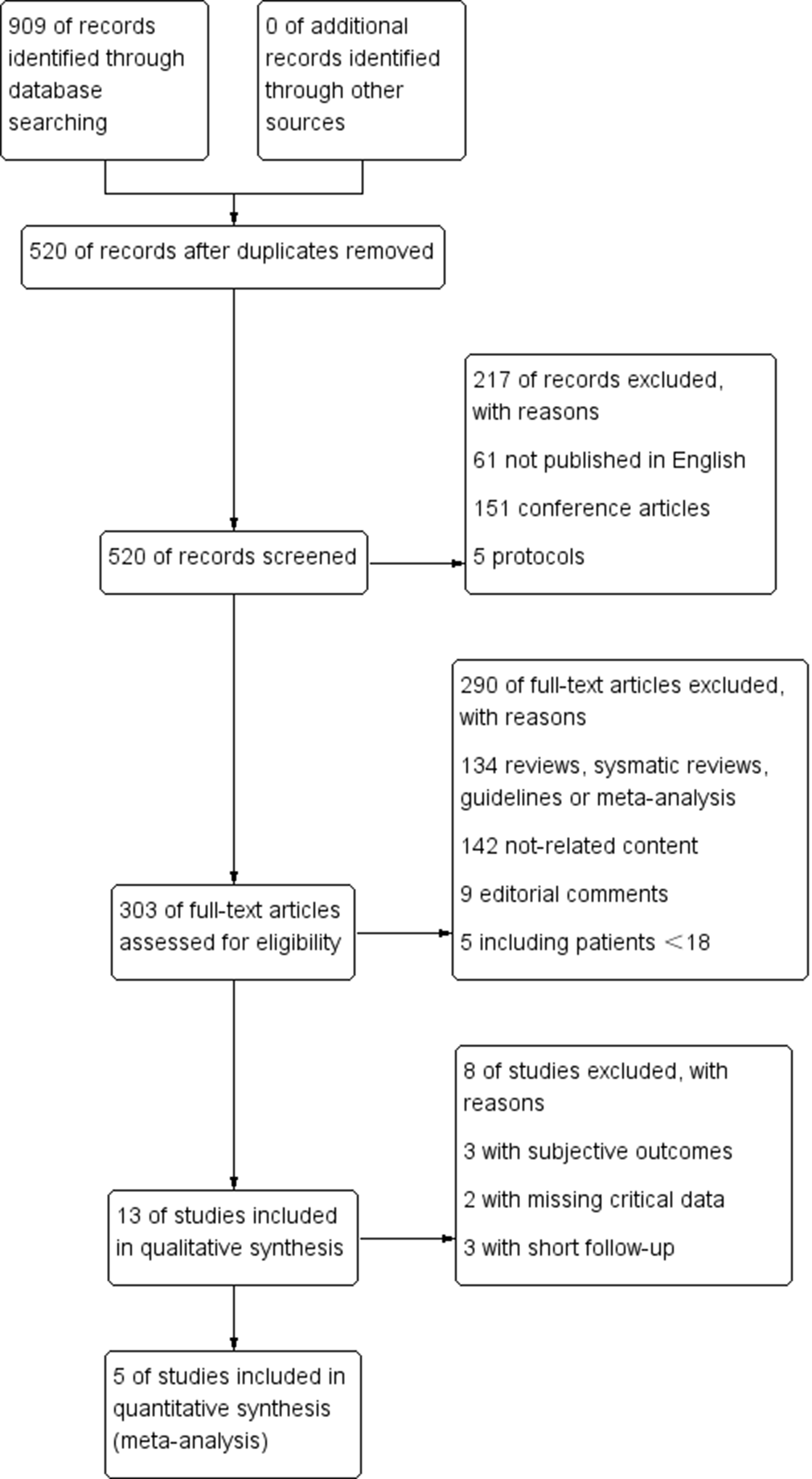

Supplement: Supplementary Figure 1 — Flow diagram of articles selection. [file Image_1.png]

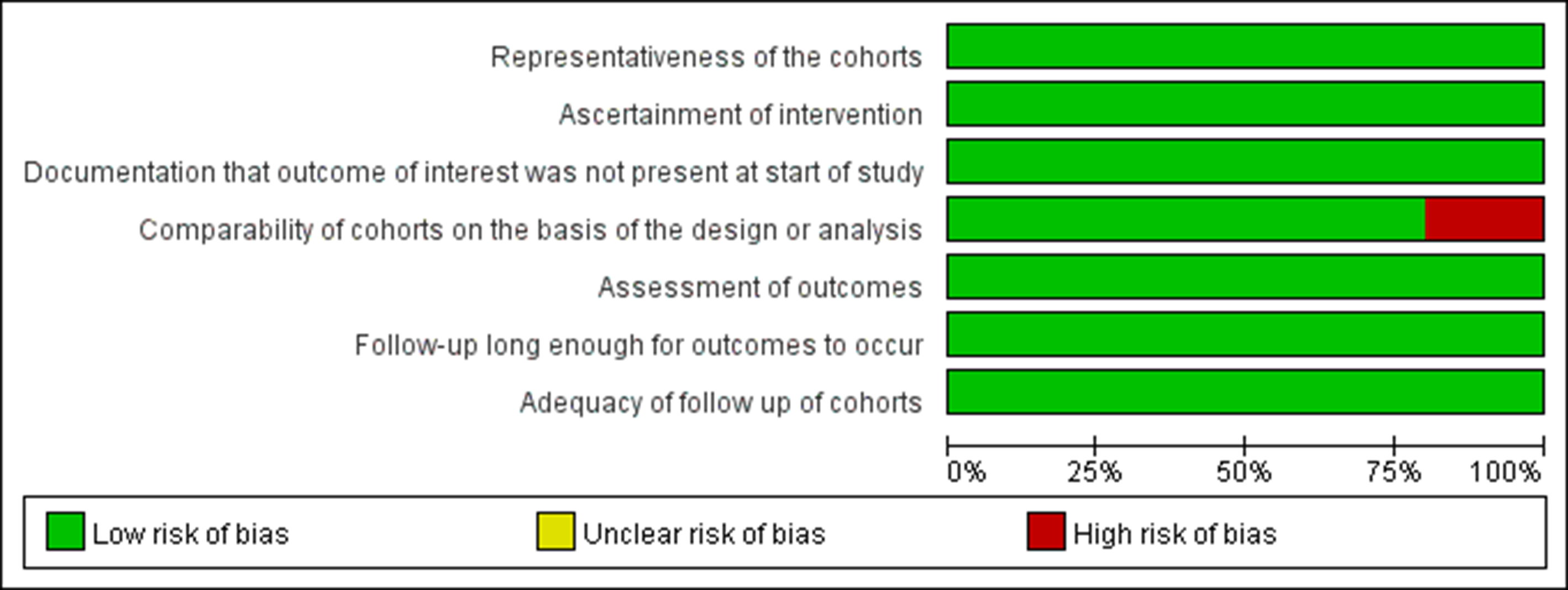

Supplement: Supplementary Figure 2 — Risk of bias graph for included studies. [file Image_2.png]

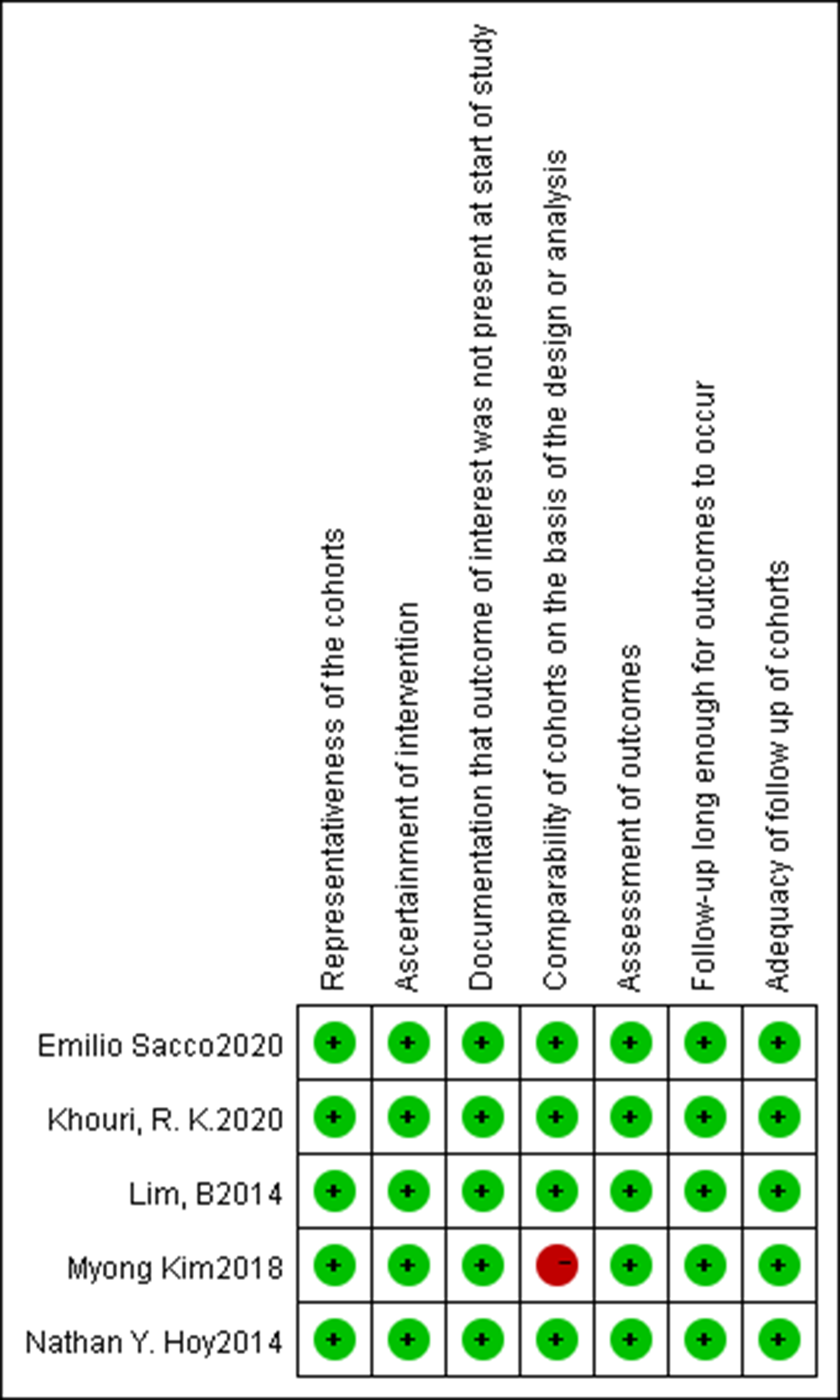

Supplement: Supplementary Figure 3 — Risk of bias summary for included studies. [file Image_3.png]

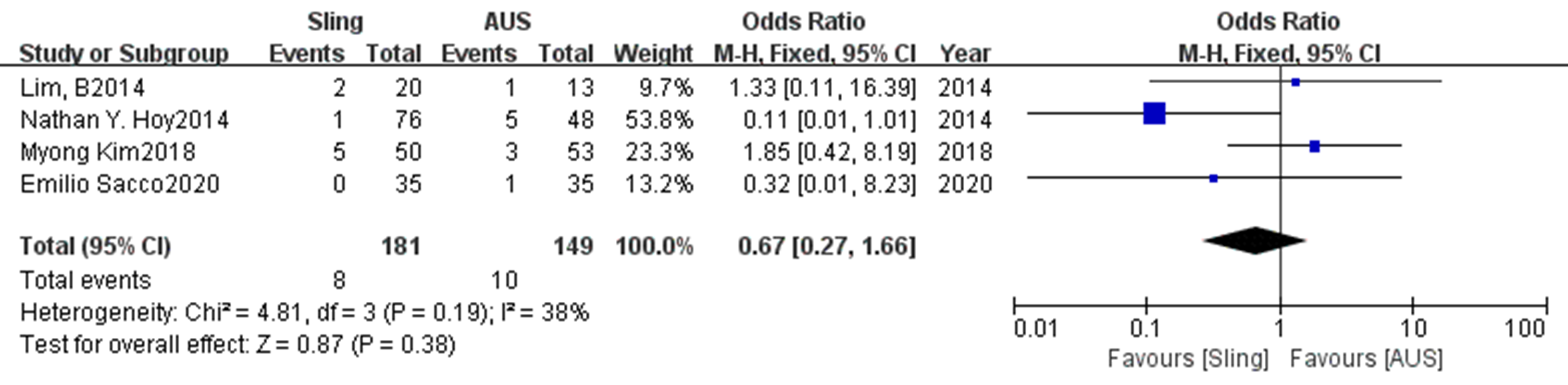

Supplement: Supplementary Figure 4 — Forest plot of comparison of infection rate for slings vs. Aus. AUS, artificial urinary sphincter. [file Image_4.png]

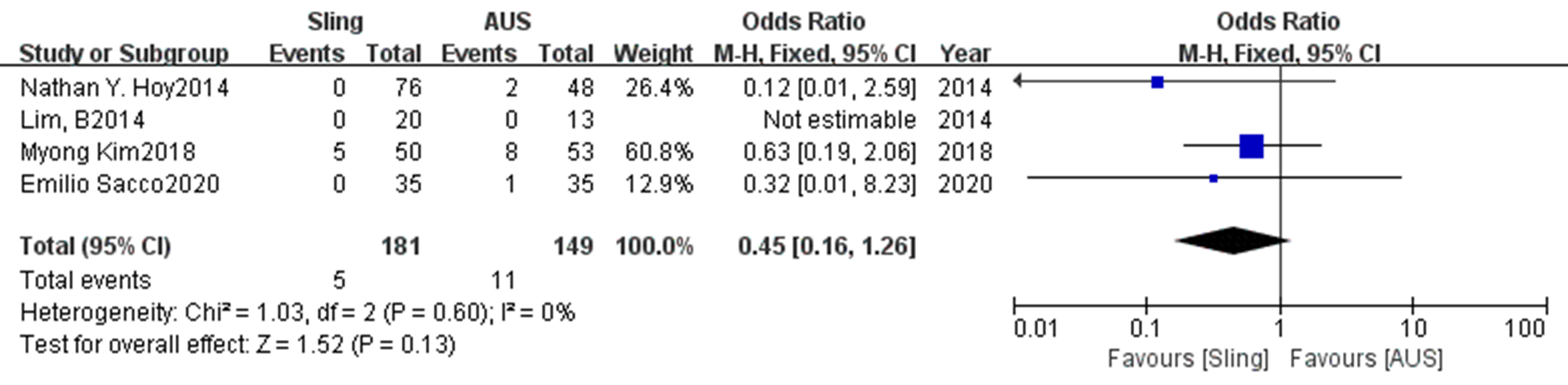

Supplement: Supplementary Figure 5 — Forest plot of comparison of erosion rate for slings vs. AUS. AUS, artificial urinary sphincter. [file Image_5.png]

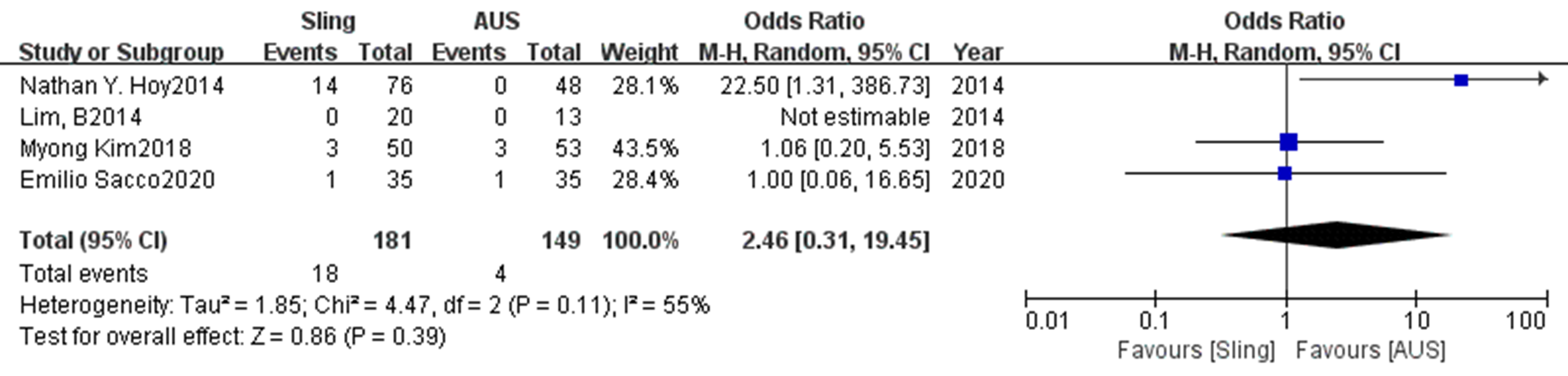

Supplement: Supplementary Figure 6 — Forest plot of comparison of acute urinary retention rate for slings vs. AUS. AUS, artificial urinary sphincter. [file Image_6.png]

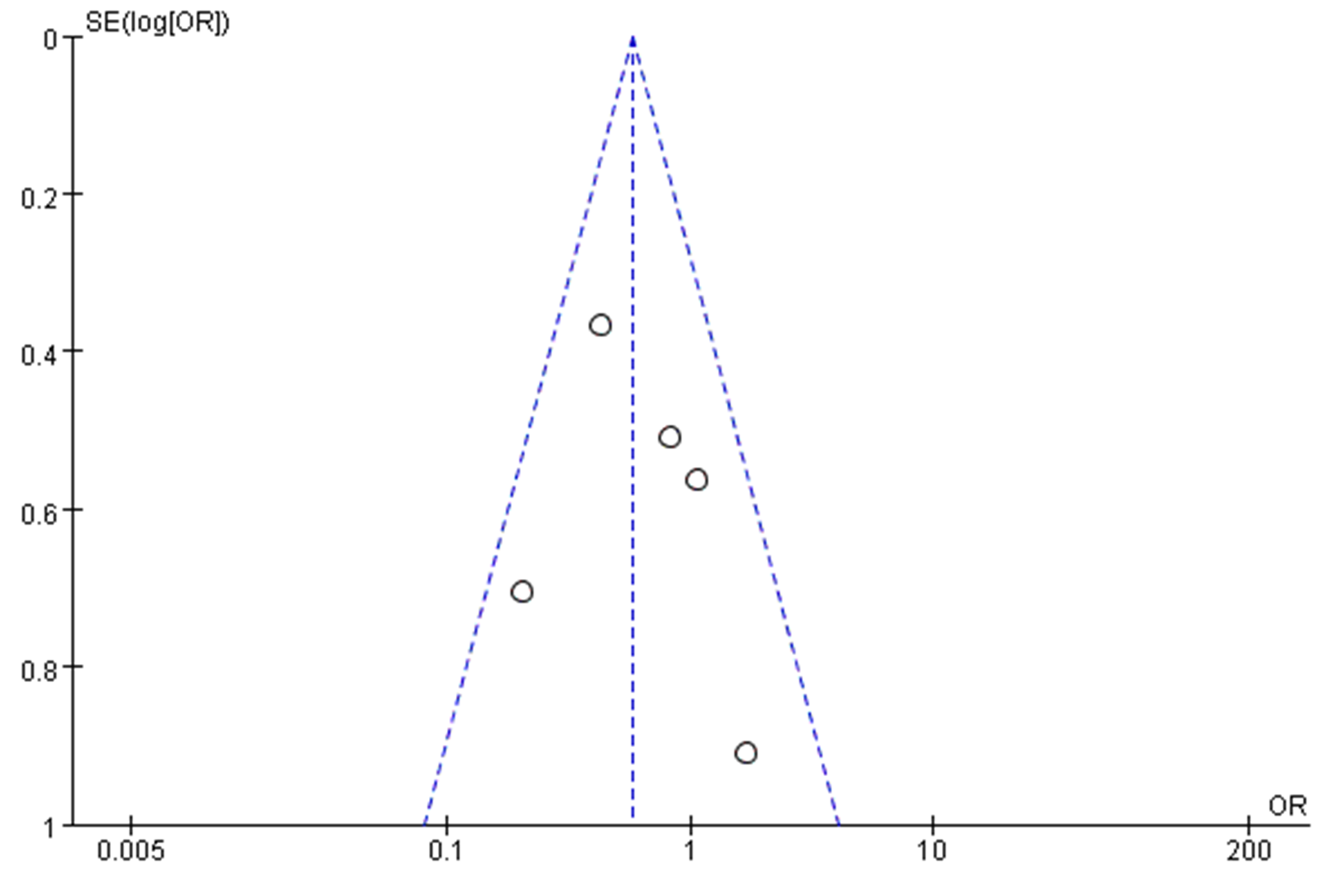

Supplement: Supplementary Figure 7 — Funnel plot of comparison of success rate for slings vs. AUS. [file Image_7.png]
